# Cartas al Editor

**Published:** 2021-06-15

Lima, 9 de marzo de 2021 

Señores

Comité Editorial

Revista *Biomédica*

Bogotá, D.C., Colombia

Estimados editores

En el suplemento 2 del volumen 40 de la revista se publicó el artículo titulado “Factores pronósticos en pacientes hospitalizados con diagnóstico de infección por SARS-CoV-2 en Bogotá, Colombia” en el cual se utilizó la prueba estadística de comparación de medias mediante la t de Student según el paradigma clásico (también llamado frecuentista) en pacientes hospitalizados con diagnóstico de infección por SARS-CoV-2 para estimar si había diferencia significativa en el promedio de días de respiración mecánica asistida entre los pacientes con enfermedad grave y aquellos que no la presentaron ^1^. En ese contexto, expongo aquí un ejemplo de un nuevo análisis bayesiano ^2^ con los datos del valor de t de -6.046 y los tamaños muestrales de 71 y 33.

En ciencias de la salud, se recomienda la replicación de los estudios con base en las pruebas de significación, con el fin de generar datos de mayor credibilidad. Ello puede hacerse mediante la inferencia bayesiana (probabilidad de los datos al confrontar la hipótesis nula con la alterna) ^2^^,^^3^. Mediante el factor de Bayes se cuantifica el grado en que los datos respaldan una u otra hipótesis para trascender la interpretación dicotómica del rechazo o aceptación de la hipótesis nula ^2^^,^^3^. Esta alternativa metodológica permite hacer inferencias a partir de los resultados observados en una muestra o población y plantear conclusiones que van más allá del marco estudiado, cuya interpretación se basa en el esquema de clasificación de valores de Jeffreys ^4^: débil, moderado, fuerte, muy fuerte y extremo (tabla 1).

**Tabla 1:** Valores de interpretación cuantificable del factor de Bayes

>100	Extrema	Hipótesis alternativa
30+100	Muy fuerte	Hipótesis alternativa
10+30	Fuerte	Hipótesis alternativa
3,1-10	Moderado	Hipótesis alternativa
1,1-3	Débil	Hipótesis alternativa
1	0	Sin evidencia
0,3-0,9	Débil	Hipótesis nula
0,29-0,1	Moderado	Hipótesis nula
0,09-0,03	Fuerte	Hipótesis nula
0,03-0,01	Muy fuerte	Hipótesis nula
<0,01	Extrema	Hipótesis nula

Nota: creación propia según la escala de clasificación de Jeffreys (4)

Las dos interpretaciones comunes del factor de Bayes en estudios comparativos son el FB_10_ (a favor de la hipótesis alternativa de diferencia), el FB_01_ (a favor de la hipótesis nula de no diferencia) y el intervalo de confianza de 95 % ^5^. En la reevaluación bayesiana que presento aquí, ambos criterios obtuvieron los siguientes valores: FB_10_=416000, FB_01_=2,4e-06 e IC_95%_ de -0,957 a -0,436. Estos hallazgos son una evidencia extrema a favor de la hipótesis estadística alterna de diferencia de medias reportada por Motta, *et al*. ^1^.

Asimismo, se reporta el parámetro *máximo* del factor de Bayes (max FB_10_= 474181) para determinar la estabilidad de los resultados, cuyo valor similar implica una mayor coherencia de los hallazgos de la inferencia bayesiana.

El factor de Bayes es de gran utilidad en otros análisis estadísticos, entre ellos, las pruebas de significación ^6^^-^^8^. Se aconseja este método alternativo porque, en diversas investigaciones biomédicas, el tamaño de la muestra es pequeño y restringido a poblaciones clínicas específicas, lo que puede conducir a resultados controversiales debido a su limitado poder estadístico (menor probabilidad de rechazar la hipótesis nula cuando es falsa) y a una mayor prevalencia de falsos positivos o errores de tipo I ^9^.

Se ha recomendado reportar los tamaños de efecto para reforzar los valores de p. Sin embargo, aún no hay un consenso claro sobre las pautas para su interpretación, ya que dichos criterios varían entre las diversas áreas y disciplinas de la salud, y según el tipo de investigación, las medidas específicas utilizadas y las poblaciones de interés ^9^. Asimismo, se carece de un valor estándar para el área biomédica, por lo que el uso del factor de Bayes es un gran aporte metodológico en este ámbito. Es aconsejable hacer estimaciones concluyentes o superiores (FB_10_>10) para una mayor certeza de la confirmación de la hipótesis alterna.

En conclusión, la inferencia bayesiana es esencial para precisar el grado de fuerza probatoria de las hipótesis estadísticas más allá del modelo de las pruebas de significación y para tomar decisiones clínicas a partir de los resultados en futuros estudios experimentales biomédicos o estudios clínicos de impacto en la salud de los pacientes.

Cristian Antony Ramos-Vera

Área de investigación, Universidad César Vallejo, Lima, Perú

## References

[1] Motta JC, Novoa D, Gómez CC, Moreno J, Vargas L, Pérez J (2020). Factores pronósticos en pacientes hospitalizados con diagnóstico de infección por SARS-CoV-2 en Bogotá, Colombia. Biomédica.

[2] Ly A, Raj A, Etz A, Gronau QF, Wagenmakers E-J (2018). Bayesian reanalyses from summary statistics: A guide for academic consumers. Adv Meth Pract Psychol Sci.

[3] Marsmamn M, Wagenmakers EJ (2017). Bayesian benefits with JASP. Eur J Dev Psychol.

[4] Jeffreys H (1961). Theory of probability.

[5] Goss-Sampson MA (2020). Bayesian Inference in JASP: A guide for students.

[6] Quintana DS, Williams DR (2018). Bayesian alternatives for common null-hypothesis significance tests in psychiatry: A non-technical guide using JASP. BMC Psychiatry.

[7] Kelter R (2020). Bayesian alternatives to null hypothesis significance testing in biomedical research: A non-technical introduction to Bayesian inference with JASP. BMC Med Res Methodol.

[8] Ramos-Vera CA (2020). Replicación bayesiana: cuán probable es la hipótesis nula e hipótesis alterna.

[9] Brydges CR (2019). Effect size guidelines, sample size calculations, and statistical power in gerontology.

