# Supplementary material for: Cartas al Editor
Source: Biomedica. 2021 Jun 15;41(2):374–7. [Article in Spanish] (PMC8387015)
Supplement: Related texts

# Reply: Cartas al Editor

Biomédica 2021;41:374-7

Bogotá, 1 de mayo de 2021

Señores Comité Editorial

Revista *Biomédica*

Bogota, D.C., Colombia

Estimados señores:

Esta carta tiene como fin dar respuesta a un comentario que hemos recibido sobre nuestro estudio publicado en el volumen 40 de la revista sobre factores pronósticos en pacientes hospitalizados con Covid-19 ^1^. El comentario presenta un ejemplo de análisis bayesiano de una de las variables medidas en nuestro estudio (promedio de días de respiración mecánica) y su efecto sobre la gravedad de la enfermedad. Dicho análisis plantea la necesidad de usar métodos bayesianos (factor bayesiano) como método aconsejado en la investigación para interpretar el grado de certeza de la aceptación de la hipótesis alterna, así como la de replicar el análisis de la prueba de independencia, que en nuestro estudio se hizo con la t de Student con resultado significativo.

Comprendemos las ventajas de utilizar los métodos bayesianos para llegar a inferencias en la investigación. Entre las más relevantes están la posibilidad de incorporar información previa sobre un parámetro y usarla para estimaciones posteriores, la de hacer inferencias cuando el tamaño de la muestra es pequeño procediendo como se haría si se contara con tamaños de muestra grandes, y el hecho de que brinda respuestas interpretables ^2^.

A pesar de que el uso de los métodos bayesianos se ha venido extendiendo a varios de los campos que involucran el uso de la estadística, es importante señalar que no están exentos de críticas. Uno de los problemas planteados por este paradigma es su gran dependencia en la subjetividad por recurrir a la identificación de una probabilidad *a priori* establecida por el investigador ^3^. Otros inconvenientes tienen que ver con el desarrollo matemático de algunas de las pruebas métricas utilizadas, como el R al cuadrado que, según Gelman, *et al*. ^4^, representa un problema para el ajuste de modelos mediante métodos bayesianos, aspecto que aún está en proceso de mejorarse. Por último, en lo relacionado con el factor de Bayes, Morey, *et al.*^5^, plantean que este es demasiado sensible a las distribuciones a *priori* y que dichas distribuciones son difíciles de determinar.

En el contexto de nuestro estudio, encontramos las siguientes limitaciones para aplicar estos métodos:

1. La inferencia realizada con el factor de Bayes que nos presenta el autor para la variable del promedio de días de asistencia respiratoria mecánica y su efecto en la gravedad de la Covid-19, no era necesaria dado que dicha asistencia mecánica está implícita en la definición de caso grave. Por ello, en nuestro estudio no hicimos inferencia en torno a esta variable como se explica en la construcción y reporte final del modelo.
2. La inferencia bayesiana requiere conocer probabilidades *a priori*, las cuales pueden llegar a ser subjetivas, o desconocerse, por lo que debe tenerse cuidado en el momento de elegirlas para no obtener resultados sesgados. En el momento del estudio no conocíamos la probabilidad *a priori* de recibir asistencia respiratoria mecánica, dato que no debe ignorarse, ya que influye fuertemente en los resultados.

En conclusión, es de suma importancia para los investigadores saber que existen alternativas frente al análisis frecuentista y que una de las más prometedoras es la de los métodos bayesianos. El establecimiento de las diferencias y similitudes de los dos métodos, así como de sus ventajas y desventajas, proporciona a los investigadores una mirada más amplia de los métodos posibles para responder las preguntas de investigación y decidir cuál es mejor en cada contexto. También, es cierto que está creciendo ampliamente el interés por usar métodos bayesianos debido al aumento en la capacidad de los computadores de escritorio que hoy permiten llevar a cabo estos análisis.

Juan Camilo Motta

Escuela de Medicina y Ciencias de la Salud, Universidad del Rosario, Bogotá, D.C., Colombia

## References

[10] Camilo MJ, Novoa DJ, Gómez CC, Moreno JM, Vargas L, Pérez J (2020). Factores pronósticos en pacientes hospitalizados con diagnóstico de infección por SARS-CoV-2 en Bogotá, Colombia. Biomédica.

[11] Kruschke JK, Liddell TM (2018). Bayesian data analysis for newcomers. Psychon Bull Rev.

[12] Greenland S (2006). Bayesian perspectives for epidemiological research: I. Foundations and basic methods. Int J Epidemiol.

[13] Gelman A, Goodrich B, Gabry J, Vehtari A (2019). squared for Bayesian regression models. Vol. 73, American Statistician. American Statistical Association.

[14] Morey RD, Romeijn J-W, Rouder JN (2016). The philosophy of Bayes factors and the quantification of statistical evidence. J Math Psychol.
